# Assessment of immune function and prediction of survival and infection in patients with severe alcoholic hepatitis: An exploratory study

**DOI:** 10.1002/jgh3.12891

**Published:** 2023-03-21

**Authors:** Paula Boeira, Huey Tan, Euan Yates, Ashwin Dhanda

**Affiliations:** ^1^ Hepatology Research Group, Faculty of Health University of Plymouth Plymouth UK; ^2^ South West Liver Unit University Hospitals Plymouth NHS Trust Plymouth UK

**Keywords:** alcoholic hepatitis, alcohol‐related liver disease, biomarker, cytokine, infection

## Abstract

**Background and Aim:**

Alcoholic hepatitis (AH), a severe complication of long‐term alcohol misuse, has a 30% 90‐day mortality. Infections are common and associated with higher mortality. There is currently no accurate method to predict infection in these patients. We aimed to test a measure of immune function, the QuantiFERON Monitor (QFM), in predicting clinical outcomes in patients with severe AH.

**Methods:**

Peripheral blood was taken at baseline, and QFM performed according to the manufacturer's instructions. In parallel, QFM samples were analyzed with a cytokine multiplex. Clinical outcomes of mortality at 28 and 90 days and development of infection were recorded prospectively.

**Results:**

Forty‐nine patients were recruited (mean age 51, 59% male and mean discriminant function 57.8). Interferon (IFN)‐γ release measured by standard QFM was significantly higher in survivors compared to non‐survivors at 28 (102 *vs* 16 IU/mL, *P* = 0.02) and 90 days (115 *vs* 32 IU/mL; *P* = 0.046). The area under the receiver operating characteristic curve (AUROC) was 0.79 for 28‐day mortality. IFN‐γ, IL‐10, and IL‐23 release measured by multiplex were significantly lower in patients who developed a subsequent infection compared to those who did not (115 *vs* 27 IU/mL, *P* = 0.037; 457 *vs* 202 pg/mL, *P* = 0.008; and 1039 *vs* 663 pg/mL, *p* = 0.01, respectively).

**Conclusion:**

Immune dysfunction is associated with poorer outcomes in patients with severe AH. Measurement of IFN‐γ release by standard QFM accurately predicts early mortality, which can be applied to clinical practice as a biomarker of survival. Adaptation of the test to measure IL‐10 could be used as a biomarker of subsequent infection to guide clinical treatment decisions.

## Graphical abstract

It is difficult to predict who will die or get an infection with alcoholic hepatitis. QuantiFERON Monitor (QFM) is a commercial assay measuring immune function that may predict early mortality and infection in alcoholic hepatitis patients. After validation, QFM could be used to guide management of alcoholic hepatitis.

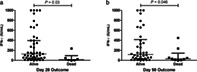

## Introduction

Alcohol use is responsible for 5.1% of the global burden of disease.^1^ Alcohol misuse can lead to alcohol‐related liver disease (ARLD), one of the most common causes of chronic liver disease and the primary cause of liver‐related mortality.^1^ Alcoholic hepatitis (AH), a severe complication of ARLD, is an acute clinical syndrome characterized by a recent onset of jaundice and coagulopathy in patients with heavy alcohol use^2^ with a 90‐day mortality of 30%.^3^ Currently, there are no proven therapies to improve long‐term survival, although corticosteroid use is associated with reduced mortality at 28 days.^3^ However, use of corticosteroids is associated with increased risk of infection and death.^4^

Furthermore, approximately 30–40% of AH patients are refractory to corticosteroid treatment.^5^, ^6^ Several clinical scores including the discriminant function (DF), Glasgow AH Score, and Model of End‐stage Liver Disease (MELD) predict short‐ and medium‐term mortality with high accuracy but do not predict the risk of infection.^6^ Biomarkers to predict corticosteroid response such as the Lille score^6^ and neutrophil–lymphocyte ratio^7^ have been proposed but, again, they do not predict the risk of infection. A novel method to stratify patients with AH at the point of clinical presentation to predict survival and the risk of infection would improve their clinical management.

AH is a condition associated with immune dysfunction, with highly activated neutrophils, monocytes, and T‐cells displaying an exhausted phenotype.^8^, ^9^, ^10^ Patients with AH are at high risk of developing infection, a major cause of mortality in this setting, which is increased by up to 70% with corticosteroid treatment.^4^, ^11^ The most common infections are urinary tract infection, bacteremia, lower respiratory tract infection, and spontaneous bacterial peritonitis (SBP).^12^, ^13^, ^14^ The high incidence of infections is associated with immune dysfunction; increased T‐cell exhaustion leads to reduced ability to prevent bacterial infection.^5^, ^10^ Despite high circulating levels of pro‐inflammatory cytokines TNF‐α, IL‐8, and IL‐17, which are anti‐pathogenic in other conditions,^15^, ^16^ immune cells are refractory to stimulation by microbial products in patients with AH, suggesting an impaired response to infection.^9^

Biomarkers to improve the detection of infection in patients with AH have been evaluated in several studies with conflicting results.^11^ C‐reactive protein and procalcitonin are elevated in all patients with AH irrespective of infection. They are predictors of mortality,^17^, ^18^ and a small cohort study showed that both biomarkers were predictors of systemic inflammatory response syndrome with infection.^19^ Other biomarkers including white cell count and serum lipopolysaccharide were not accurate in predicting infection.^12^, ^18^

QuantiFERON Monitor (QFM; QIAGEN, Dusseldorf, Germany) is a cytokine release assay designed to measure immune function by stimulating ex vivo whole blood with T‐cell receptor and toll‐like receptor (TLR) ligands, activating both innate and adaptive immune cells and measuring subsequent IFN‐γ expression by enzyme‐linked immunosorbent assay (ELISA). It has been developed from the well‐established QuantiFERON TB Gold assay, which stimulates whole blood with tuberculous antigens^20^ and is recommended for diagnosis of tuberculosis by the World Health Organization. It is widely used in clinical laboratories, which have established protocols and equipment; results can be available within 24 h. In the current study, we investigate the accuracy of QFM, a commercially available assay of immune function, to predict mortality and infection in patients with AH.

## Methods

### Patient selection

The study received ethical approval from the National Health Service Health Research Authority (ref: 15/LO/1501). All patients provided written informed consent, or where lacking capacity, assent was obtained from a personal or nominated consultee. The study was conducted in accordance with the International Conference on Harmonization Good Clinical Practice for Research and the Declaration of Helsinki. Consecutive patients presenting to University Hospitals Plymouth NHS Trust with severe AH diagnosed according to the National Institute for Alcohol Abuse and Alcoholism^2^ and with DF of 32 or more were recruited to the study. A peripheral blood sample was obtained as soon as possible after hospital admission and before any specific treatment for AH was given. Routine clinical and laboratory parameters were obtained at baseline and at Days 28 and 90. Survival and development of infection were documented at each study visit.

### Laboratory assays

QFM was performed according the manufacturer's instructions. In brief, 1 mL of peripheral blood was drawn directly into each of two pre‐vacuumed lithium heparin collection tubes. A lyosphere, as supplied by the manufacturer, containing anti‐CD3 and R848 was added to one tube (positive tube) and mixed by gentle inversion. Both tubes were maintained upright at 37°C in an incubator with 5% CO_2_ for 18–24 h. Both tubes were then centrifuged at 2000*g* for 15 min. Serum was removed by pipette and stored in two aliquots each at −80°C for further analysis. One aliquot was defrosted, and IFN‐γ concentration was determined by the ELISA kit supplied as part of the QFM kit. Samples with values above the upper limit of detection (1000 IU/mL) were assigned a value of 1000 IU/mL. The second aliquot was tested for 11 analytes (IFN‐γ, TNF‐α, IL‐6, IL‐10, IL‐1β, CCL20, IL‐4, IL‐17, IL‐2, IL‐12, and IL‐23) by multiplex. Samples were run in duplicate, and the mean value was used for analysis.

The multiplex assay (R&D Systems, Oxford, UK) was performed according to manufacturer's instructions and analyzed using a Luminex 200 system (Luminex Corporation, Austin, Texas, USA). Protein concentration was quantified using supplied standards of known concentrations.

### Statistical analysis

Normality distribution in continuous data was assessed using the Kolmogorov–Smirnov test. Continuous variables that were normally distributed were summarized using mean and standard deviation (SD) and those not normally distributed with median and interquartile range (IQR). Groups that were not normally distributed were compared using the Mann–Whitney *U*‐test. Categorical variables were described using frequencies and percentages and compared using the Fisher exact test. Correlations were performed using Pearson's correlation coefficient. Accuracy of variables in predicting a specific outcome was determined using the area under the receiver operating characteristic (AUROC). All statistical analyses were performed using IBM SPSS Statistics version 25 (IBM, Armonk, New York, USA).

## Results

### Patient characteristics

Forty‐nine patients (59% male, mean age 51, mean DF 57.8, mean MELD 22.4) with AH were recruited (Table 1). Twelve out of the 49 patients (24%) received corticosteroid treatment during their hospital admission. Those who did not had contraindications to corticosteroids due to infection, active gastrointestinal bleeding, or acute kidney injury. Patient characteristics and AH severity (DF and MELD) were similar between patients who received corticosteroids and those who did not (Table S1).

**Table 1:** Patient characteristics in whole cohort and separated by survival status at Day 90

	All (*n* = 49)	Alive at Day 90 (*n* = 40)	Dead at Day 90 (*n* = 9)
Age	51.0 (10.7)	50.4 (11.1)	53.4 (8.8)
Gender (% male)	59%	58%	63%
DF	57.8 (31.9)	56.4 (33.3)	63.8 (25.6)
MELD	22.4 (5.3)	21.8 (5.4)	24.9 (4.8)
Bilirubin (μmol/L)	209 (139)	202 (138)	242 (148)
Albumin (g/L)	29 (5.2)	29 (5.5)	28 (3.8)
INR	1.5 (0.4)	1.5 (0.4)	1.6 (0.3)
White blood count ×10^9^/L	9.8 (5.1)	9.5 (4.9)	11.0 (0.8)
Acute kidney injury, *n*	0	0	0
Hepatic encephalopathy, *n*	8	5	3
Spontaneous bacterial peritonitis, *n*	1	0	1
Acute on chronic liver failure, *n*	0	0	0
D28 mortality	6 (12%)		
D90 mortality	9 (18%)		

Continuous variables are presented as mean (standard deviation).

Six (12%) patients died within 28 days and 9 died (18.4%) within 90 days. Age, DF, and MELD were not significantly different between survivors and non‐survivors at day 28 or 90 (Table 1). All patients who received corticosteroids survived to 90 days.

Thirteen (27%) patients had a documented infection at baseline (62% chest, 23% urine, 15% SBP) and 12 other patients (25%) developed a subsequent (incident) infection during their hospital stay (50% chest, 42% urine, 8% bacteraemia).

### QFM may be associated with survival

**Figure 1:**
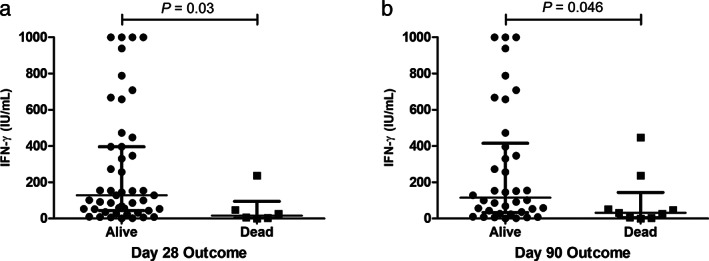
Dotplot of QFM IFN‐y concentration in survivors and non‐survivors at (a) Day 28 and (b) Day 90. Bars show median and IQR (*n* = 49).

IFN‐γ, measured by QFM according to manufacturer's instructions, was higher in patients who survived compared to those who died within 28 (102 *vs* 16 IU/mL, *P* = 0.02; Fig. 1) and 90 days (115 *vs* 32 IU/mL; *P* = 0.046; Fig. 1 and Table 2). Overall, in all 49 patients, the mean IFN‐γ release was 239 IU/mL (SD 309) and median 92 IU/mL (IQR 321). IFN‐γ measured by multiplex assay was not statistically significantly different in survivors *versus* non‐survivors at day 28 (12 672 *vs* 6307 pg/mL; *P* = 0.24) but it was at day 90 (13 640 *vs* 4260 pg/mL; *P* = 0.03; Table 2). The other 10 analytes measured by multiplex were not different between survivors and non‐survivors at either Day 28 or 90 (Table 2).

**Table 2:** Median (IQR) of cytokines in patients who survived and died within 90 days

	Alive at Day 90 (*n* = 40)	Dead at Day 90 (*n* = 9)	*P*‐value
IFN‐γ (IU/mL)^†^	115 (382)	32 (140)	**0.046** *
IFN‐γ (pg/mL)^†^	5763 (19083)	1575 (6985)	**0.046** *
IFN‐γ (pg/mL)	13 640 (17665)	4260 (10061)	**0.03** *
TNF‐α (pg/mL)	7039 (4777)	6563 (3019)	0.68
IL‐6 (pg/mL)	10 170 (3193)	9667 (6514)	0.40
IL‐10 (pg/mL)	390 (421)	402 (618)	1.00
IL‐1β (pg/mL)	5941 (5118)	7032 (4373)	0.78
CCL20 (pg/mL)	8952 (3581)	7004 (3443)	0.14
IL‐4 (pg/mL)	88 (23)	90 (19)	0.66
IL‐17 (pg/mL)	296 (446)	103 (396)	0.12
IL‐2 (pg/mL)	79 (208)	148 (220)	0.95
IL‐12 (pg/mL)	307 (32)	312 (60)	0.98
IL‐23 (pg/mL)	963 (539)	858 (666)	0.25

^†^ IFN‐γ release after standard QFM; results shown in IU/mL and pg/mL.

* Statistically significant *P* < 0.05.

IFN‐γ measured by standard QFM was not associated with clinical parameters including severity scores (DF [*r* = −0.06] and MELD [*r* = −0.01]), total white cell count (*r* = 0.23), lymphocytes (*r* = 0.13), or neutrophils (*r* = 0.23).

All patients who received corticosteroids survived to 90 days and had median IFN‐γ release by QFM of 86 IU/mL. IFN‐γ measured by standard QFM and all analytes measured by multiplex were similar between patients who received or did not receive corticosteroids.

The AUROC for 28‐day mortality was 0.79 (95% confidence interval 0.61–0.98) for IFN‐γ and 0.57 and 0.62 for DF and MELD, respectively. IFN‐γ concentration was dichotomized at the optimum threshold for specificity and sensitivity as determined by the Youden point at 52 IU/mL. At this threshold, the sensitivity and specificity to predict 28‐day mortality was 0.83 and 0.68, respectively, and for 90‐day mortality was 0.78 and 0.71, respectively.

### Cytokine release and infection

**Figure 2:**
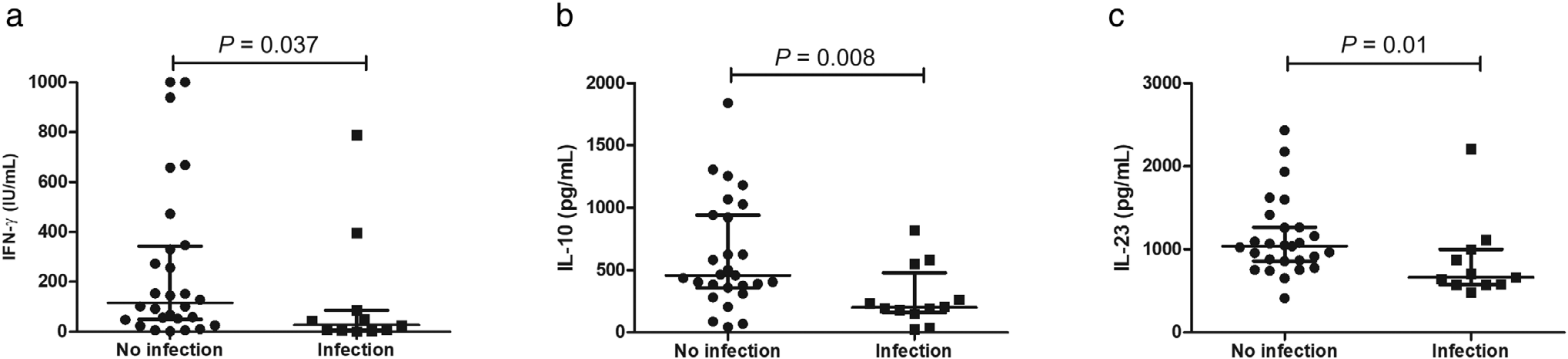
IFN‐γ release by standard QFM (a) and IL‐10 (b) and IL‐23 (c) release measured by cytokine multiplex in patients who subsequently did and did not develop and infection. Bars show median with IQR (*n* = 49).

Low expression of IFN‐γ (by standard QFM and multiplex), IL‐10, and IL‐23 was associated with incident infection (115 *vs* 27 IU/mL, *P* = 0.037; 457 *vs* 202 pg/mL, *P* = 0.008; and 1039 *vs* 663 pg/mL, *P* = 0.01, respectively; Fig. 2; Table 3). Incident infection was not associated with corticosteroid treatment (*P* = 0.25). There was no association between any cytokine expression and infection at baseline.

**Table 3:** Median (IQR) of cytokines released by patients who did and did not develop a subsequent infection

	No infection^†^ (*n* = 37)	Infection (*n* = 12)	*P*‐value
IFN‐γ (IU/mL)^‡^	115 (293)	27 (79)	**0.037** *
IFN‐γ (pg/mL)^‡^	5763 (14648)	1350 (3954)	**0.045** *
IFN‐γ (pg/mL)	14 970 (19068)	5807 (13834)	**0.045** *
TNF‐α (pg/mL)	7122 (2509)	5574 (4177)	0.25
IL‐6 (pg/mL)	10 170 (2995)	9575 (5302)	0.60
IL‐10 (pg/mL)	457 (583)	202 (320)	**0.008** *
IL‐1β (pg/mL)	7097 (3777)	5176 (5606)	0.13
CCL20 (pg/mL)	7785 (3416)	9056 (3758)	0.71
IL‐4 (pg/mL)	90 (18)	78 (18)	0.28
IL‐17 (pg/mL)	355 (346)	128 (386)	0.29
IL‐2 (pg/mL)	76 (188)	108 (186)	1.00
IL‐12 (pg/mL)	312 (25)	297 (15)	0.07
IL‐23 (pg/mL)	1039 (408)	663 (426)	**0.01**

^†^ The no‐infection group also included patients who had an infection at baseline.

^‡^ IFN‐γ release after standard QFM; results shown in IU/mL and pg/mL.

* Statistically significant *P* < 0.05.

## Discussion

The present study is the first to report that impairment in immune function measured by cytokine release assay is associated with infection and mortality, which supports the existing literature on immune dysfunction in severe AH. The functional QFM assay demonstrates that patients with a more robust immune response had reduced incident infection and improved survival, identifying IFN‐γ, IL‐10, and IL‐23 as possible biomarkers of outcome.

IL‐10 is a multifunctional cytokine known to have both anti‐ and pro‐inflammatory effects depending on the immune environment.^21^ In comparison to healthy controls, patients with AH were found to have much lower circulating IL‐10.^22^, ^23^ However, these studies did not examine the relationship between IL‐10 and infection in AH patients. In contrast to the current study, a T‐cell cytokine release assay predicted survival in AH patients, with increased production of anti‐inflammatory interleukin (IL)‐10 associated with death.^24^ Another study found that polymorphisms in genes resulting in high TNF‐α and low IL‐10 production were associated with higher rates of sepsis in patients with AH.^25^ In the context of sepsis, IL‐10 is higher in non‐survivors compared to survivors^26^ and polymorphisms associated with high IL‐10 production are associated with higher risk of bacterial sepsis.^27^ These conflicting results show the challenges of interpreting the biological relevance of IL‐10 in AH. Further confirmatory studies are needed to evaluate the association between IL‐10 and infection in AH.

IL‐23 is a pro‐inflammatory cytokine produced by monocytes, macrophages, and dendritic cells, which is important for Th17 cell survival and function.^28^ Th17 cells provide protection against pathogens, with effectiveness against extracellular bacteria and fungi.^29^ These roles in the protection against infection explain why low IL‐23 release on peripheral blood stimulation is associated with the development of infection in patients with AH. However, IL‐23 was not an independent predictor of subsequent infection on multivariate analysis, suggesting that its downregulation may simply reflect a systemic attenuated cytokine response.

IFN‐γ is essential in modulating an array of immune responses, including the regulation of bacterial infections. Levels of IFN‐γ were found to be reduced in the serum of patients with chronic ARLD.^30^ In a cytokine release assay, levels of T‐cell production of IFN‐γ was predictive of 90‐day mortality in AH patients.^24^ Furthermore, IFN‐γ‐induced genes were significantly decreased in severe AH patients and these patients were highly susceptible to infections.^29^ In the present study, IFN‐γ measured by standard QFM was strongly associated with 28‐ and 90‐day mortality. However, when measured by multiplex, the observed differences failed to meet statistical significance at Day 28. This is explained by higher intra‐test variability of approximately 10% as well as use of different measurement units in the multiplex with broader ranges of values and standard deviations.

Our findings suggest that the levels of IL‐10 and IFN‐γ release may be promising biomarkers to predict subsequent infection in AH patients. This may be useful to optimize prophylactic antibiotic therapy in those at the highest risk of infection, especially in light of recent trial data suggesting that empirical antibiotics in addition to corticosteroids in unselected patients with severe AH does not reduce mortality.^31^

We acknowledge that this is a single‐center observational cohort study and requires validation in other centers. However, our patient population is representative of patients with AH in the United Kingdom, with similar severity scores and survival rates as those reported in the STOPAH trial.^3^ Furthermore, patients were recruited consecutively and followed up prospectively in sufficient number to permit an analysis according to clinical outcomes of mortality and infection.

## Conclusions

This study shows that the QFM assay may be useful to predict clinical outcomes of mortality and infection in patients with AH. The QFM test is a commercially available assay based on the well‐established QuantiFERON TB Gold platform, with results available within 24 h. It can easily be performed in clinical laboratories and, after further validation, has the potential to be implemented in clinical pathways to guide treatment decisions such as initiation of prophylactic antibiotics.

## Supporting information


**Data S1.** Supporting information.
