# Supplementary material for: Assessment of immune function and prediction of survival and infection in patients with severe alcoholic hepatitis: An exploratory study
Source: JGH Open. 2023 Mar 21;7(4):286–90. doi: 10.1002/jgh3.12891 (PMC10134762; doi:10.1002/jgh3.12891)
Supplement: Supplementary file 1 — Data S1. Supporting information. [file JGH3-7-286-s001.docx]

**Supplementary material**

Supplementary table 1. Patient characteristics separated by treatment with corticosteroids. Variables are presented as mean (standard deviation).

|  | No corticosteroids (n=37) | Corticosteroids (n=12) | p-value |
| --- | --- | --- | --- |
| DF | 53.7 (27.2) | 70.4 (42.3) | 0.12 |
| MELD | 21.9 (5.5) | 23/8 (4.8) | 0.29 |
| Bilirubin (µmol/L) | 118 (132) | 207 (134) | 0.05 |
| Albumin (g/L) | 32.1 (6.3) | 30.8 (7.0) | 0.55 |
| INR | 1.44 (0.39) | 1.58 (0.39) | 0.28 |
| White blood count x10^9^/L | 8.1 (4.8) | 8.0 (3.0) | 0.94 |
| Creatinine (µmol//L) | 76.7 (34.0) | 58.9 (12.4) | 0.09 |
